# Early Feeding Strategies for the Larviculture of the Vermiculated Angelfish *Chaetodontoplus mesoleucus*: The Key Role of Copepods

**DOI:** 10.3390/ani15162437

**Published:** 2025-08-20

**Authors:** Yu-Hsuan Sun, Yu-Ru Lin, Hung-Yen Hsieh, Pei-Jie Meng

**Affiliations:** 1 Graduate Institute of Marine Biology, College of Environment Studies and Oceanography, National Dong Hwa University, Hualien 974301, Taiwan; angel750508@gmail.com; 2 Department of Aquaculture, College of Life Science, National Taiwan Ocean University, Keelung 202301, Taiwan; 3 Taiwan Ocean Research Institute (TORI), National Institutes of Applied Research, Kaohsiung 85243, Taiwan; 4 Department of Oceanography, National Sun Yat-Sen University, Kaohsiung 80424, Taiwan; 5 Research Center for Critical Issues, Academia Sinica, Taipei 11529, Taiwan

**Keywords:** *Chaetodontoplus mesoleucus*, *Euplotes* sp., *Bestiolina coreana*, copepods, larviculture, live prey, angelfish aquaculture

## Abstract

The captive breeding of marine ornamental fish with specialized larval requirements—such as *Chaetodontoplus mesoleucus*—remains a major bottleneck in aquaculture, largely due to the lack of techniques tailored to their unique morphological and nutritional needs. The global marine ornamental aquaculture market is valued at approximately USD 2.15 billion annually; however, only around 10% of marine ornamental species are currently supplied through captive breeding, highlighting a substantial technological gap. The artificial propagation of *C. mesoleucus* is particularly challenging due to the species’ small mouth gape and high nutritional demands during early development. To address this issue, we evaluated the effects of three live-prey types—*Euplotes* sp., *Brachionus* sp., and *Bestiolina coreana*—as well as a mixed diet containing all three, on larval performance. From 3 days post-hatch, larvae were fed each prey type at equal densities (15–20 individuals/mL), and water quality was carefully maintained to minimize external influences. Survival and total length were assessed at 14 dph. At the end of the trial, the mixed-diet group showed the highest survival rate (36.2 ± 5.6%), whereas larvae fed only *B. coreana* exhibited the greatest total length (7.4 ± 1.2 mm) and a high metamorphosis rate (97.8%). These findings demonstrate that prey selection significantly influences the early survival and growth in *C. mesoleucus* larvae and highlight the critical role of copepods in promoting growth performance. However, as larval biomass was not quantified, the findings should be interpreted with caution, and future studies incorporating biomass assessments are needed to draw more conclusive inferences. The successful mass rearing of this species supports the feasibility of captive production to reduce wild harvesting, protect coral-reef biodiversity, and promote sustainable ornamental aquaculture.

## Simple Summary

Marine ornamental fish exhibit a narrow larval gape that limits prey selection and high sensitivity to water-quality fluctuations, making successful larviculture exceptionally challenging. Consequently, nearly 90% are still harvested directly from coral-reef ecosystems for the aquarium trade. This practice places pressure on natural populations and fragile reef habitats. To support more-sustainable approaches, our study focused on developing captive breeding techniques for a popular ornamental angelfish species, *Chaetodontoplus mesoleucus*. We tested different small zooplankton commonly used as food in early fish rearing to find the best combination for survival and growth. The results showed that offering a mix of these live-prey sources helped more young fish to survive, while feeding only copepods led to better growth. These findings not only contribute to the successful breeding of reef fish in captivity but also help to reduce the need for wild collection. Promoting the use of copepods as a key dietary component is essential for improving breeding outcomes and protecting coral-reef biodiversity in the long term.

## 1. Introduction

In recent years, the global marine ornamental aquarium industry has grown significantly, with an estimated annual trade value exceeding US$1 billion [1]. The EU is a major importer, recording around €24 million annually from 2014 to 2021, involving over 1400 species [2]. Among them, Pomacanthidae ranked second, comprising 18% of imports [1]. Despite this growth, over 90% of marine ornamental species are still wild-caught [3,4]. Aquaculture is seen as a solution, yet technical challenges—such as replicating marine conditions and accommodating species-specific requirements—limit progress. Advances in larval rearing, live feeds, and breeding protocols are essential for reducing wild collection and ensuring sustainability [3,5].

*Chaetodontoplus mesoleucus*, the Vermiculated angelfish, is prized in the marine ornamental trade for its vivid coloration and adaptability. Currently, all specimens supplied to the aquarium market are wild-caught, exposing them to intense capture and transport stress and resulting in high mortality. Although early trials in 1995 managed to rear larvae only to 5 dph, full-life-cycle culture under controlled conditions remained unrealized [6]. Across Pomacanthidae, only 12 angelfish species have completed full captive breeding, and scalable protocols are still lacking [7]. Notably, our team has now achieved the first successful settlement of *C. mesoleucus*, with newly hatched larvae that measured 2.50 ± 0.07 mm in total length. At 3 dph, larvae were actively swimming, the anus had opened, gape height had increased to 0.411 ± 0.024 mm, and they were ready to feed [8]. Given the high value of small-mouth Pomacanthidae in the ornamental fish trade, we selected this species to compare various zooplankton feeds and develop robust captive-breeding protocols. Establishing a reliable closed-cycle breeding program for *C. mesoleucus* could substantially reduce wild-capture losses and help meet the growing market demand.

Live prey play a pivotal role in the successful rearing of marine fish larvae [9]. Rotifers (*Brachionus* sp.) and brine shrimp (*Artemia* sp.) are commonly utilized in commercial aquaculture due to their ease of culture and availability [10]. However, their application in marine ornamental fish larviculture often encounters limitations, such as inadequate nutritional profiles and their suboptimal size for first-feeding larvae, leading to reduced survival and growth rates [1,10,11]. Consequently, researchers have explored alternative live prey, with copepods emerging as a superior option. Copepods offer enhanced nutritional value, including higher levels of essential fatty acids like DHA and EPA, and better mimic the natural diet of marine fish larvae, resulting in improved survival, growth, and pigmentation [9,10]. Nonetheless, mass-producing copepods at scale remains challenging. For species with particularly small mouth openings and underdeveloped feeding capabilities at first feeding, even copepod nauplii may be too large [12]. In such cases, ciliates, due to their smaller size and slower movement, have been proposed as initial live prey [10]. While ciliates can be ingested by early-stage larvae, studies have shown that they may not sufficiently support larval growth and development, highlighting the need for further research into their nutritional adequacy and potential supplementation strategies [11].

Different fish larvae demonstrate species-specific preferences for live-prey organisms [13,14]. Even live feeds generally regarded as optimal based on standard nutritional or physical parameters may not be universally accepted or suitable across all species. Consequently, empirical assessments are essential to determine the most appropriate and effective prey organisms tailored to the biological characteristics of each species. Given the range of factors that influence prey selection in marine fish larvae—including mouth gape limitations, prey motility, and sensory detectability—we selected three live-prey types that fall within the ingestible size range of newly hatched *Chaetodontoplus mesoleucus* larvae: *Euplotes* sp. (a ciliate), *Brachionus* sp. (a rotifer), and *Bestiolina coreana* (a copepod). These prey organisms were offered at equal densities to ensure a standardized comparison of larval responses. All three prey types were pre-sieved to ensure comparable sizes and were administered either individually or as a mixed diet to *C. mesoleucus* larvae. This experimental design allowed us to assess not only the individual suitability of each prey type, but also to compare outcomes between single-species and combined-species feeding regimes. By conducting these comparisons across developmental time points, we aimed to determine whether different prey types exert distinct stage-specific effects on larval survival, growth, or metamorphosis. Moreover, the mixed-diet group enabled us to investigate potential prey–prey interactions, such as behavioral interference, prey preference shifts, or selective feeding dynamics.

In doing so, this study seeks to identify optimal early feeding strategies that can improve larval rearing success and ultimately support the development of sustainable captive breeding protocols for this high-value ornamental species.

## 2. Materials and Methods

### 2.1. Egg Collection

The broodstock comprised captive-bred progeny of wild *C. mesoleucus*—originating from multiple wild broodstock pairings—that had been maintained in a recirculating aquaculture system for approximately four years. Fertilized eggs were collected on a near-daily basis from March 2023 through December 2024. The broodstock group consisted of one male (9–10 cm total length, TL) and three females (7–8 cmTL). Each day after 17:00, fertilized eggs were collected at the initial water outlet of the recirculating aquaculture system (RAS) using an 80-mesh net, and retrieved before 20:00. Collected eggs were transferred into beakers and allowed to settle; non-viable, sinking eggs were discarded. Only buoyant, healthy fertilized eggs were retained and examined under a dissecting microscope to confirm normal fertilization and development. Confirmed healthy eggs were then gently blotted to remove excess water, weighed, counted, and utilized for further experimental use.

### 2.2. Live-Prey Organism Culture

*Nannochloropsis oculata*, *Tetraselmis chui*, and *Isochrysis galbana* were cultured in 20 L transparent containers with f/2 medium under semi-continuous conditions [13]. Cultures were maintained at 26–30 °C under a 14L:10D photoperiod (6000–8000 Lux) with gentle aeration. Peak algal density was typically reached within 7–10 days for harvest or propagation.

*Euplotes* sp. was cultured semi-continuously in 100 L tanks with sterilized seawater supplemented with 0.2 mL/L fish hydrolysate, kept at 26–30 °C without aeration and in the absence of algal supplementation. Cultures peaked in 3–5 days. S-type *Brachionus* sp. were cultured in 500 L tanks. Seawater was bleached, neutralized with sodium thiosulfate, and supplemented with commercial fertilizer (Hua Bao No. 2; containing 20.0% total nitrogen [4.0% ammonium nitrogen, 4.0% nitrate nitrogen], 20.0% water-soluble P_2_O_5_, and 20.5% water-soluble K_2_O) at 1g ton^−1^. *N. oculata* was added and gently aerated. After 5–7 days, rotifers were introduced and reared at 24–30 °C. Peak densities occurred after 7–10 days. *B. coreana* (strain: NTOU-Best.-1) was cultured in 1000 L tanks. Seawater was bleached and neutralized, then enriched with *I. galbana* at 10^5^–10^6^ cells/mL before copepod inoculation. Aeration and algal concentrations were maintained daily to ensure stable feeding. Peak densities were reached in 7–10 days at 24–30 °C.

### 2.3. Feeding Experiment

Fertilized eggs, all obtained from a single spawn on the same day, were pooled into a single container and gently mixed to achieve a uniform suspension. From this homogenate, subsamples were randomly withdrawn and counted, and 500 eggs were allocated to each 500 L fiberglass tank. Each treatment comprised three replicate tanks using eggs from that same batch. The experimental culture water was prepared two days prior by filtering natural seawater and adjusting it to a temperature of 27.0 ± 0.5 °C and a salinity of 33–34 ppt. The water was inoculated with a 1:1:1 mixture of *N. oculata*, *T. chui*, and *I. galbana* at a total concentration of 10^4^–10^5^ cells/mL. Gentle aeration was provided to ensure water circulation, and a photoperiod of 12 h L:12 h D was maintained. Water quality parameters, including salinity (33–34 ppt), pH (7.9–8.4), and dissolved oxygen (≥80% saturation), were monitored and regulated similarly to the broodstock RAS system; these items were measured with a multiparameter meter (YSI ProQUATRO, Yellow Springs, OH, USA). Beginning at 3 dph, a 24 h continuous flow-through system was implemented, with a daily water exchange of approximately 200 L to maintain NH_4_-N and NO_2_-N levels below 0.1 ppm, measured with a portable spectrophotometer (Hach DR900, Loveland, CO, USA) [15]. To prevent larval loss, the outlet was equipped with a 100-mesh net with gentle aeration, creating hydrodynamic turbulence and mesh retention. Larval survival was recorded daily, and survival rates were calculated based on the number of surviving larvae at each time point. At 14 days post-hatch, 15 larvae per treatment were first randomly sampled for total-length measurement; simultaneously, morphological observations were conducted to record the number of individuals that had completed metamorphosis, and the metamorphosis rate was calculated accordingly. Metamorphosis was defined as reaching the postflexion stage at 14 dph, following the method outlined in [8] and the stage definitions of the authors in [16]. The survival rates and metamorphosis rates were calculated using the following equation:

$$Survival\;Rate\;(\%)=(\frac{Number\;of\;surviving\;larvae\;at\;1\;to\;14\;dph}{Number\;of\;hatched\;larvae\;at\;0\;dph})\times 100$$

$$Metamorphosis\;Rate\;(\%)=(\frac{Number\;of\;postflexion\;larvae\;at\;14\;dph}{Number\;of\;larvae\;at14\;dph})\times 100$$

The experiment was divided into four feeding treatments. Body length ranges of the live-prey species were measured in this study. For treatment A, larvae were provided exclusively with *Euplotes* sp. (approximately 70–90 μm) at a density of 15–20 ind/mL, beginning at 2 dph and continuing through 14 dph. For treatment B, larvae were fed *Brachionus* sp., (approximately 90–180 μm) also at a density of 15–20 ind/mL, from 2 to 14 dph. Treatment C involved feeding larvae with *B. coreana* nauplii (approximately 90–120 μm) from 2 to 8 dph, followed by the introduction of *B. coreana* copepodites (approximately 100–160 μm) from 9 to 14 dph, also at a density of 15–20 ind/mL. For treatment D, larvae were initially provided with a mixture of *Euplotes* sp., *Brachionus* sp., and *B. coreana* nauplii, each at a density of 5–7 ind/mL, from 2 to 8 dph. From 9 to 14 dph, only *Brachionus* sp. and *B. coreana* nauplii and copepodites were supplied, each at a density of 8–10 ind/mL. The prey densities used in this experiment were selected based on established practices in the larval rearing of marine fish species, with similar ranges (15–20 ind/mL) having been reported to support optimal survival and feeding performance [17,18]. Feed density was measured four times daily (08:00, 11:00, 14:00, and 17:00) and replenished as necessary to maintain the designated levels in each treatment. Due to the significant size difference between *B. coreana* nauplii (<0.09 mm) and adults (0.85–0.96 mm) [19,20], a size-sorting procedure was conducted to ensure prey were of suitable size for larval ingestion. For treatments C and D, from 2 to 7 dph, *B. coreana* were collected using a 200-mesh net (74 μm), and individuals retained by a 150-mesh net (104 μm) were removed. From 8 to 10 dph, *B. coreana* were collected with a 200-mesh net and sorted by excluding those retained by a 120-mesh net (125 μm). From 11 to 14 dph, the larvae were fed *B. coreana* collected using a 150-mesh net, again removing larger individuals retained on a 120-mesh net to maintain an appropriate prey size. Each treatment was conducted in triplicate, and the experimental period lasted 14 days, encompassing the critical early-larval phase during which mortality rates are highest. To facilitate accurate counting, live-prey samples were first chilled on ice for several minutes to reduce motility. After gentle mixing, three 0.5 mL subsamples were randomly collected and placed onto glass slides for microscopic observation. Prey organisms in each subsample were counted under a stereomicroscope, and the average value was used to estimate the prey density (ind/mL). Based on the calculated density and the volume of water in each experimental group, the total number of prey required was determined, and the corresponding volume of live-prey culture was added to each tank to achieve the designated feeding density. The quantification of live-prey densities within the larval tanks followed the methodology described by Gatesoupe and Luguet [21]. Details of the treatments and feeding regimes are summarized in Table 1.

### 2.4. Statistical Analysis

The data are presented as means ± standard deviations (SDs), with 95% confidence intervals (95% CIs) also calculated. The following dependent variables were analyzed to evaluate larval performance across different feeding treatments: survival rate (%), total length (mm) at 14 dph, and metamorphosis rate (%). Normality and homogeneity of variance were assessed by the Shapiro–Wilk and Levene tests, respectively. One-way ANOVA followed by a Tukey’s honest significant difference post hoc test was conducted at α = 0.05, using GraphPad Prism 10.4.0.

## 3. Results

**Figure 1:**
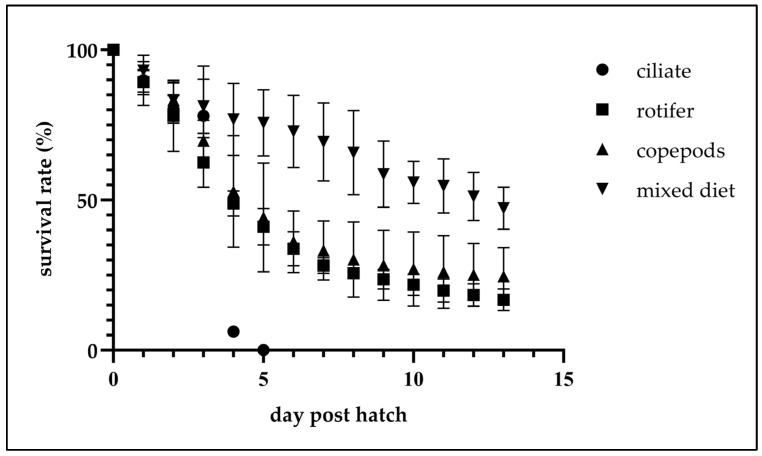
Survival rates (%) of *C. mesoleucus larvae* in different feeding experiments. Data are presented as mean *±* SD.

**Figure 2:**
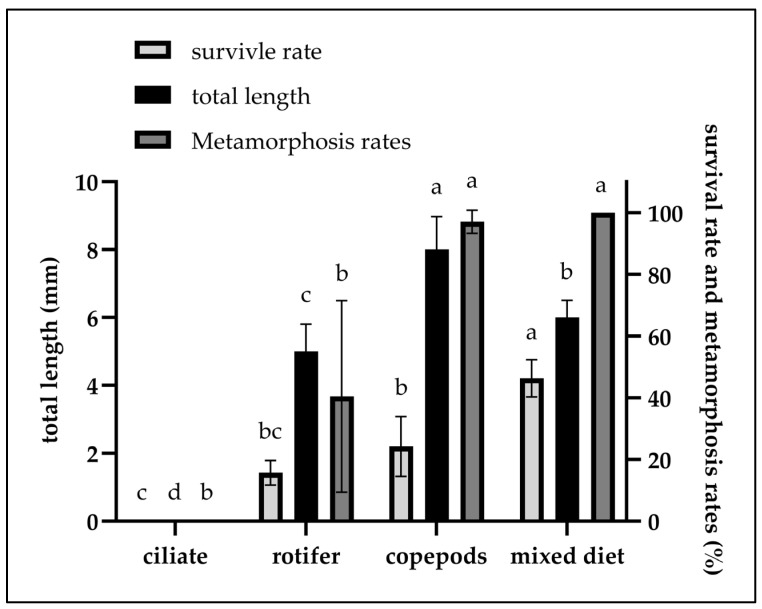
Survival rates (%), total length (mm) (*n* = 15), and metamorphosis rates (%) (*n* = 15) of *C. mesoleucus larvae* in different feeding experiments at 14 dph. Data are presented as mean *±* SD. Different superscripts indicate significant difference between treatments (*p* < 0.05).

The number of fertilized eggs of *C. mesoleucus* was about 2128 ± 215 per gram (*n* = 5). At a water temperature range of 26.6–27.3 °C, the initial hatching rates of treatments A, B, C, and D were 77.4 ± 4.4%, 74.0 ± 7.8%, 77.2 ± 0.5%, and 78.0 ± 1.1%, respectively, with no significant differences observed among treatments (*p >* 0.05); at 3 dph, survival rates were 59.9 ± 9.1%, 46.1 ± 7.1%, 53.9 ± 5.6%, and 63.3 ± 7.7% across the same respective treatments, also without significant differences (*p >* 0.05). However, at 4 dph, mass mortality was experienced with treatment A, with survival dropping sharply to 4.8 ± 1.4%, which was significantly lower than with treatments B, C, or D (36.4 ± 6.6%, 40.0 ± 14.4% and 60.0 ± 10.3%) (*p <* 0.05). Complete mortality was observed with treatment A at 5 dph. On this day, survival rates for treatments B, C, and D were 30.7 ± 7.4%, 30.7 ± 7.4%, and 59.1 ± 9.1%, respectively. On the same day, significant differences were found between treatments A and D when compared to the other treatments (*p <* 0.05), while no significant difference was detected between treatments B and C (*p >* 0.05); In these treatment groups, the survival rate declined gradually over time, representing normal attrition. Detailed daily survival-rate data are presented in Table 2 and illustrated in Figure 1. At 14 dph, the highest survival rate was recorded with treatment D (36.2 ± 5.6%), which was significantly higher than with treatments C (18.7 ± 7.6%), B (11.8 ± 3.8%) and A (0%) (*p <* 0.05), and the results of treatment groups C and B did not significantly differ from each other (*p >* 0.05) (Figure 2). Total length measurements at the end of the trial revealed significant differences among treatment groups B (4.9 ± 0.8 mm), C (7.4 ± 1.2 mm), and D (6.5 ± 0.5 mm) (*p <* 0.05) (*n* = 15) (Figure 2). And, the metamorphosis rate with treatment B (40.4 ± 31.0%) was significantly lower than with treatments C (97.8 ± 3.8%) and D (100.0 ± 0%) (*n* = 15) (*p <* 0.05) (Figure 2).

Treatment group D achieved the highest survival (36.2 ± 5.6%) and complete metamorphosis (100%; *n* = 15) at 14 dph, whereas treatment group A experienced total mortality by 5 dph. Treatment C yielded moderate survival (18.7 ± 7.6%), high metamorphosis (97.8 ± 3.8%), and the greatest total length (7.4 ± 1.2 mm; *n* = 15). Treatment group B performed poorest in both survival (11.8 ± 3.8%) and metamorphosis (40.4 ± 31.0%). These results underscore the superior rearing conditions of treatment D and the growth advantage of treatment C.

## 4. Discussion

Prey size critically determines ingestion success in marine fish larvae [22,23]. At 3 dph, *C. mesoleucus* larvae exhibit a mouth gape of 0.411 ± 0.024 mm [8], corresponding to an ingestible prey size of 0.082–0.206 mm [24]. Because all test prey (*Euplotes* sp., *B. coreana*, and *Brachionus* sp.) fall within this range, survival and growth differences reflect prey motility and nutrition rather than a gape limitation.

*Euplotes* sp. is widely recognized as an important first-feeding live prey for marine fish larvae owing to its small body size and proven ingestibility [25,26], but its low nutritional content (40% protein, 5.6% total fatty acids; EPA 0.17%, DHA 0.10% of dry mass) limits long-term survival [26,27,28,29]. Multiple studies [30,31,32] have reported that larvae fare poorly on an exclusive *Euplotes* diet but much better when ciliates are combined with other prey. For example, *Elacatinus figaro* and *Gobiosoma evelynae* showed improved survival with ciliate-supplemented rotifers [30,31], and *Chaetodontoplus septentrionalis* completed metamorphosis only on a mixed diet of *Euplotes*, rotifers, and copepods [32]. These findings underscore the role of ciliates as a “bridge” prey, facilitating the transition from endogenous to exogenous feeding. However, long-term growth and survival depend on introducing higher-value prey, especially copepods. [26,30,31].

Rotifers are among the most ubiquitous live prey in aquaculture because of their environmental resilience, ease of mass culture, and slow swimming speed, which facilitates first feeding [14]. Although they support early development in many commercially important species, rotifers alone cannot satisfy the coral-reef and marine ornamental fishes. Naturally, rotifers contain 3.1% n-3 HUFAs; enrichment raises protein to 52–59% and lipids to 7–13% of dry mass [32]. Nonetheless, their limited levels of essential fatty acids lead to poorer survival, reduced growth, and delayed metamorphosis compared to other live feeds [33]. Marine larvae in particular require high levels of DHA to optimize survival and stress tolerance [34], and inadequate nutrition results in stunted somatic growth [35,36]. Exclusive rotifer feeding through 30 dph leads to essential fatty-acid-deficiency-driven growth retardation, a smaller body size, and reduced survival; for instance, *Labrus bergylta* larvae sustained on rotifers alone exhibited significantly lower survival, stunted growth, and increased deformity rates compared to those fed copepods [11,35,37,38].

Copepods have repeatedly been shown to enhance survival and growth in marine fish larvae [11,32,36,39]. In the wild, their active swimming and associated chemical cues trigger strong feeding responses in larvae, making copepods a primary natural prey [34,35]. Under culture conditions, most marine species also prefer copepods over rotifers, though preferences may vary by taxon [25]. Copepods deliver superior nutrition (50% protein, 10–20% lipids, high ω-3 HUFA) essential for survival and stress resilience [14,37]. In natural populations, copepod lipids range 6.9–22.5% of dry mass (EPA 8.3–24.6%, DHA 13.9–42.3%) [40]. Early work demonstrated that feeding copepod nauplii instead of rotifers significantly improved survival in *Sparus aurata*, *Serranus* spp., and various ornamental species [34], a pattern confirmed across *additional taxa* [34,35]. For example, *Gadus morhua* larvae exhibited accelerated growth when switched to larger copepod prey at 25 dph [39].

Multiple prey types enhance larval performance by offering varied sizes and balanced nutrients, thereby promoting growth and minimizing deformities [22,38]. For example, mixed-prey protocols have successfully reared *Labroides dimidiatus* [15] and increased survival in *Pseudochromis fridmani* [18], *Synchiropus splendidus* [41], and *Plectropomus leopardus* [42]. Likewise, *Amphiprion clarkii* larvae fed a combination of copepods exhibited significantly greater growth and survival than those on single-prey diets [43]. Specifically, combining slow-swimming ciliates, enriched rotifers, and ω-3 HUFA-rich copepods between 2–5 dph increases feeding success, elevates survival by 20–30% at 10 dph, and stabilizes growth [14,44,45].

In this study, we compared four live-prey regimens—*Euplotes* sp. (ciliates), *B. coreana* (copepods), *Brachionus* sp. (rotifers), and a mixed diet—on *C. mesoleucus* larval survival and development through 14 dph. At 3 dph, survival did not differ among treatment groups, yet the rank order (mixed diet > *Euplotes* sp. > *B. coreana* > *Brachionus* sp.) was already established and persisted. Larvae at this stage confront a “point of no return” bottleneck due exhaustion of endogenous nutrient reserves [46], which the mixed diet alleviates by supplying slow-moving *Euplotes* sp. for immediate nutrient uptake. However, larvae fed *Euplotes* only suffered a sharp mortality event on 4 dph and were completely depleted by 5 dph, illustrating the limitations of single-prey ciliates. Between 3 and 6 dph, survival in the groups fed *B. coreana* or *Brachionus* declined rapidly before stabilizing, whereas the mixed-diet group exhibited no major mortality pulses, confirming that prey diversity buffers larvae by offering multiple feeding options. This feeding preference has also been confirmed in numerous studies [14,44,45]. Although final survival under *B. coreana* was lower than in the mixed-diet treatment group and comparable to *Brachionus* sp. alone, *B.-coreana*-fed larvae attained the greatest total length, followed by those fed a mixed diet and then rotifers, and achieved 97.8 ± 4.6% metamorphosis—far surpassing the 40.4 ± 31.0% metamorphosis in rotifer-fed larvae.

Copepods deliver both potent feeding stimuli and superior nutrition. Notably, *B. coreana* nauplii are small for a copepod species—ideal for the restricted gape of Pomacanthidae—and can be mass-produced under controlled conditions, making them an optimal first-feeding live prey. Despite the survival advantage observed with the mixed diet, the slightly reduced growth of larvae compared to those fed *B. coreana* highlights the need to transition from *Euplotes* sp. and *Brachionus* sp. to *B. coreana* after initial feeding. We therefore recommend reducing the *Euplotes* sp. and *Brachionus* density post-7 dph and increasing *B. coreana* availability to maximize both survival and larval growth, providing a clear strategy for improving marine ornamental fish larviculture.

This study confirms the benefit of a diverse live-prey regimen for the early larval rearing of *C. mesoleucus*. However, ciliates remain notoriously difficult to culture consistently, and no reliable mass-production protocol exists. Their cultivation is subject to many uncontrolled variables, and ciliate nutrition composition fluctuates with environmental conditions. Future work should incorporate biomass assessments and develop scalable culture methods to better define the requirements of key live feeds. Ultimately, integrating biomass assessments with improved live-feed culture technologies will be pivotal for establishing reliable captive-rearing protocols that both strengthen the marine ornamental aquarium industry and contribute to the long-term conservation of coral-reef ecosystems.

## 5. Conclusions

The results of our study indicate that in *C. mesoleucus*, prey size alone does not ensure successful larval rearing—nutrition must also be adequate to prevent mortality. In the artificial propagation of *C. mesoleucus*, offering newly hatched larvae a diverse array of small prey—ciliates, rotifers, and nauplii of copepods—facilitates successful initial feeding. Gradually increasing the proportion of copepods from 7 dph onwards, until they fully replace ciliates and rotifers, proved to be an effective feeding strategy. This approach highlights the importance of survival, growth, and metamorphosis rates, supporting successful larval development, although the absence of biomass measurements limits the conclusiveness of the results. Because biomass is a key parameter for evaluating food efficiency in fisheries and aquaculture studies, its inclusion should represent a critical improvement in future work. Refining feeding protocols with such assessments, alongside advances in nutritional enrichment and scalable live-prey production, will ultimately enhance rearing efficiency while reducing reliance on wild-caught stocks and supporting the sustainability of the marine ornamental aquarium industry.

## Figures and Tables

**Table 1:** Daily rations of live-prey regimes fed to each experimental treatment in the larval-rearing stage.

Treatment	2–7 dph	8–10 dph	11–14 dph
A	E (15–20 ind/mL)	E (15–20 ind/mL)	E (15–20 ind/mL)
B	R (15–20 ind/mL)	R (15–20 ind/mL)	R (15–20 ind/mL)
C	Cn (15–20 ind/mL)	Cn + Cc (15–20 ind/mL)	Cc (15–20 ind/mL)
D	E (5–7 ind/mL) + R (5–7 ind/mL) + Cn (5–7 ind/mL)	R (8–10 ind/mL) + Cn + Cc (8–10 ind/mL)	R (8–10 ind/mL) + Cc (8–10 ind/mL)

All treatments in triplicate. E. *Euplotes* sp.; R. *Brachionus* sp.; Cn. *B. coreana* nauplii; Cc. *B. coreana* copepodites.

**Table 2:** Survival rates of *C. mesoleucus* larvae in feeding experiments. Data are presented as mean ± SD. Different superscripts indicate significant difference between treatments (*p* < 0.05).

Treatment	0 dph	1 dph	2 dph	3 dph	4 dph	5 dph	6 dph	7 dph	8 dph	9 dph	10 dph	11 dph	12 dph	13 dph	14 dph
A	100.0 ^a^	89.9 ± 8.4 ^a^	80.2 ± 4.6 ^a^	78.0 ± 16.6 ^a^	6.2 ± 1.6 ^b^	0.0 ^c^									
B	100.0 ^a^	89.3 ± 4.2 ^a^	78.0 ± 11.9 ^a^	62.5 ± 8.3 ^a^	48.9 ± 4.2 ^a^	41.1 ± 6.1 ^b^	33.8 ± 5.7 ^b^	28.2 ± 2.6 ^b^	25.6 ± 1.6 ^b^	23.6 ± 3.3 ^b^	21.8 ± 3.5 ^b^	19.9 ± 3.9 ^b^	18.4 ± 3.8 ^b^	16.8 ± 3.6 ^b^	15.7 ± 4.0 ^b^
C	100.0 ^a^	89.5 ± 3.6 ^a^	83.3 ± 5.8 ^a^	69.8 ± 6.8 ^a^	52.9 ± 18.6 ^a^	44.2 ± 18.1 ^b^	36.1 ± 10.3 ^b^	33.2 ± 9.8 ^b^	30.2 ± 12.5 ^b^	28.3 ± 11.9 ^b^	27.0 ± 12.7 ^b^	26.0 ± 12.1 ^b^	25.1 ± 10.4 ^b^	24.6 ± 9.5 ^b^	24.2 ± 9.7 ^b^
D	100.0 ^a^	93.1 ± 3.4 ^a^	83.3 ± 6.9 ^a^	81.2 ± 9.9 ^a^	76.9 ± 12.7 ^a^	75.7 ± 11.3 ^a^	72.9 ± 12.6 ^a^	69.4 ± 11.0 ^a^	65.8 ± 14.2 ^a^	58.6 ± 11.3 ^a^	55.9 ± 7.7 ^a^	54.7 ± 9.0 ^a^	51.2 ± 8.9 ^a^	47.3 ± 7.8 ^a^	46.3 ± 6.5 ^a^

All treatments in triplicate. Treatment A: E. *Euplotes* sp.; treatment B: R. *Brachionus* sp.; treatment C: *B. coreana*; treatment D: mixed diet.

## Data Availability

The original contributions presented in this study are included in the article. Further inquiries can be directed to the corresponding author(s).
